# The potential pitfalls of synovial sarcoma mimicking intraneural ganglion cyst: A case report and literature review

**DOI:** 10.1016/j.ijscr.2023.107916

**Published:** 2023-02-03

**Authors:** Abdulaziz Saleh Almodumeegh, Mohammad Talal Nouri, Hatan Mortada, Mohammed Olaish AlHasan, Faisal M. Obeid, Abdullah E. Kattan

**Affiliations:** a Medical College, Al-Imam Muhammad ibn Saud University, Saudi Arabia; b College of Medicine, King Saud University, Riyadh, Saudi Arabia; c Division of Plastic Surgery, Department of Surgery, King Saud University Medical City, King Saud University, Riyadh, Saudi Arabia; d Department of Plastic Surgery & Burn Unit, King Saud Medical City, Riyadh, Saudi Arabia; e Faculty of Medicine, Omdurman Islamic University, Khartoum, Sudan; f Department of Surgery, College of Medicine, Imam Mohammad Ibn Saud Islamic University, Riyadh, Saudi Arabia; g Division of Plastic Surgery, Department of Surgery, College of Medicine, King Saud University, Riyadh, Saudi Arabia

**Keywords:** Ganglion cyst, Case report, Synovial sarcoma, Surgical excision, Pitfall

## Abstract

**Introduction and importance:**

Synovial sarcoma is a rare soft tissue sarcoma (STS) that accounts for 5–10 % of all STS. Synovial sarcoma of the peripheral nerve is very rare, with only 26 cases reported in the literature. Hence, this case report describes an unusual presentation of synovial sarcoma mimicking intraneural ganglion cysts and a literature review.

**Presentation of case:**

We describe a 36-year-old female who presented to our clinic complaining of left leg pain for six years. MRI was done, which revealed a cystic lesion. With an impression of intraneural ganglion cyst versus nerve sheath tumor of the common peroneal nerve. The patient underwent exploration surgery and mass excision. The mass was sent for histopathology following excision, where the results indicated monophasic synovial sarcoma. An additional surgery, an epineurectomy of the common peroneal nerve and tumor bed excision, was followed by adjuvant chemotherapy with a Doxorubicin-based regimen. Following surgery, our patient's neurological symptoms improved.

**Clinical discussion:**

The mainstay of treatment in synovial sarcoma is surgical excision with a Doxorubicin-based regimen of chemotherapy and/or radiotherapy based on tumor characteristics. Tumors smaller than 5 cm in MRI usually show homogenous enhancement and can be mistaken for benign tumors. Hence, a biopsy should be done before surgery to avoid misdiagnosis.

**Conclusion:**

Even though it is extremely rare, synovial sarcoma of the lower extremity should be considered when a painful swelling of the lower leg is associated with a long duration of symptoms. Such lesions are best managed by surgical excision and postoperative chemotherapy.

## Highlights

• Synovial sarcoma is a rare soft tissue sarcoma (STS) that accounts for 5–10 % of all STS.
• That most commonly arises in the lower limbs near the knee joint.
• Synovial sarcoma of the lower extremity should be considered when a painful swelling of the lower leg is associated with a long duration of symptoms
• STS lesions are best managed by surgical excision and histopathology examination.

## 1 Introduction

*Sarcomas* are malignancies that account for 1 % of human cancers [1]. That divide into soft tissue sarcoma (STS) and sarcoma of the bone [2]. Synovial sarcoma is a rare soft tissue sarcoma that accounts for 5–10 % of all STS [3], [4], [5]. With an incidence in Europe of 4–5/100,000/year [6]. That most commonly arises in the lower limbs near the knee joint [7], [8]. However, Synovial sarcoma of the peripheral nerve is very rare, with only 26 cases reported in the literature and only 3 in the peroneal nerve [9]. In contrast to other STS, synovial sarcoma is a slow-growing tumor with an insidious onset that usually leads to delayed diagnosis by up to two years [10]. Similarly, to synovial sarcoma, intraneural ganglion cysts also most commonly arise in the knee near the fibular head involving the peroneal nerve [11]. Moreover, both can look similar in magnetic resonance imaging (MRI) [12]. Hence, this case report describes an unusual presentation of synovial sarcoma mimicking intraneural ganglion cysts and a literature review. Based on SCARE 2020 guidelines, the case report was written [12].

## 2 Presentation of case

We describe a 36-year-old female, not known to have any chronic medical condition. The patient presented to our clinic complaining of left leg pain and swelling for six years. The patient sought medical attention as the lump grew larger over time and worsened within the past year.

On examination, the patient was vitally stable with a tender leg lump measuring 4 × 4 cm in the left aspect of the leg below the knee joint at the common peroneal nerve distribution with positive Tinel's sign & no skin changes or discharge.

The sensation was 4/5 at the dorsum of the left foot (superficial peroneal nerve), 1/5 at the first web space of the left foot (deep peroneal nerve), and normal at the lateral left foot (Sural Nerve). Power on Dorsiflexion was 3/5, 3/5 on eversion & normal on both toe extension & plantar flexion. The lab investigation requested was unremarkable.

**Fig. 1:**
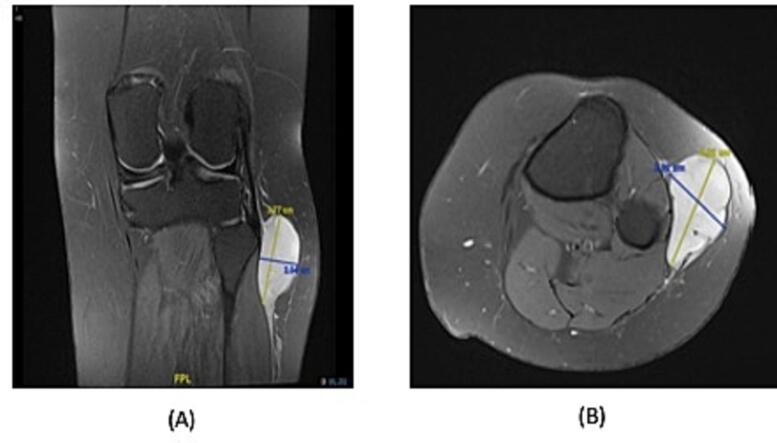
This is a contrast-enhanced MRI of the lesion. (A) The coronal view revealed a cystic lesion approximately 3.7 × 2 cm in the lateral head of gastrocnemius, adjacent to the common peroneal nerve. (B) The axial view revealed a cystic lesion approximately 4 × 2.9 cm in the lateral head of gastrocnemius.

**Fig. 2:**
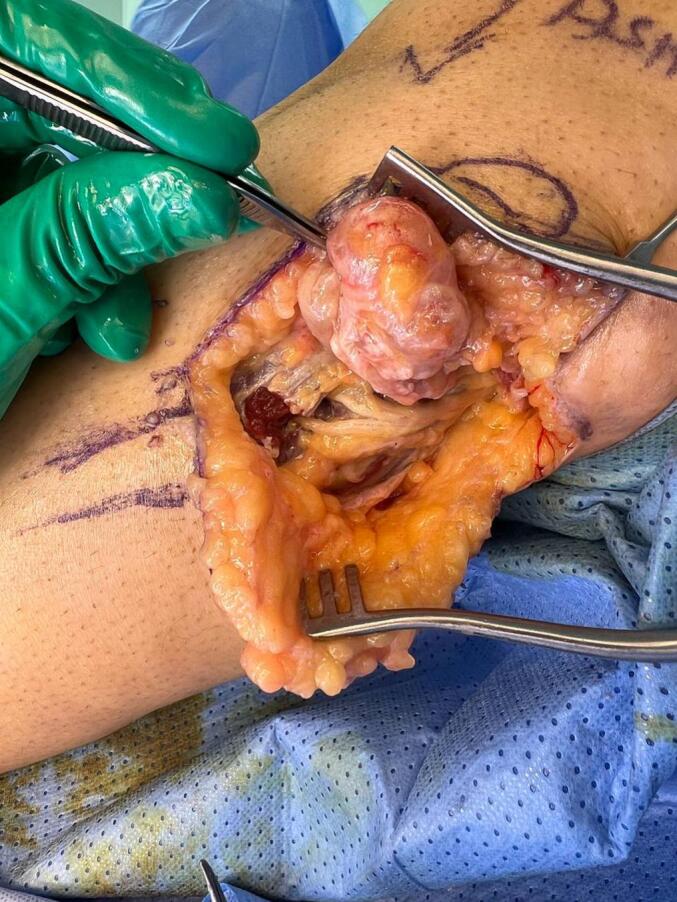
The common peroneal nerve is shown in relation to the mass in an intraoperative image of the lesion.

MRI was done, which revealed a cystic lesion in relation to the biceps femoris and lateral head of gastrocnemius with heterogeneous enhancement. The lesion measured approximately 4.4 × 5.4 × 2 cm in AP, craniocaudal and transverse diameter, respectively (Fig. 1). With an impression of intraneural ganglion cyst versus nerve sheath tumor of the common peroneal nerve. The patient underwent surgery in which we visualized and identified the common peroneal nerve intact. Its divisions into superficial and deep were also intact (Fig. 2). The nerve was released from the overlying fascia proximally and distally. As we dissected the mass, we kept the cutaneous branches of the nerve intact, cauterized the stalk, and established hemostasis.

**Fig. 3:**
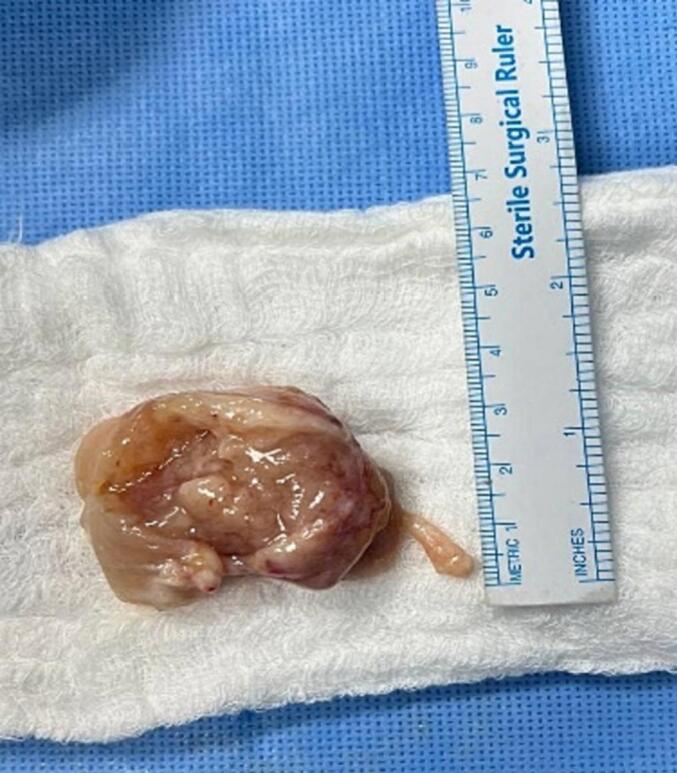
An intraoperative image showing the mass after it has been excised.

The mass was sent for histopathology following excision (Fig. 3), where the results indicated monophasic synovial sarcoma. Immunohistochemical studies have been done, and they showed that the malignant cells are strongly positive for BCL2, focally and strongly positive for EMA, focally positive for CKpan, and weak focally positive for CD99. Immunostain for Caldesmon is positive, and S100 showed weak focal positive staining. The proliferative marker Ki67 is positive in 20 % of tumor cells nuclei. CD34. SMA and Desmin are negative. Surgical margins were involved.

Following surgery, our patient's neurological symptoms have dramatically improved. An examination in the clinic showed that the wound had healed well, with no pain at the tumor site, swelling, redness, or discharge. No tenderness, sensation restored completely & muscle power improved significantly (4/5). Within one day of the operation, the patient was able to walk. The case was discussed in the tumor board, and since surgical margins were involved, another surgery and a pre-operation radiotherapy were planned. The patient completed 5000 centigray in 25 fractions with no interruptions. Then the patient underwent left lateral knee synovial sarcoma wide excision with a 2 cm safe margin. The tumor bed was removed as one unit with the underlying fascia, and common peroneal nerve epineurectomy. A tumor bed was sent for histopathology and the result came back negative for malignancy. An adjuvant chemotherapy was decided for the patient for the following reasons, size is more than 5 cm, histology of synovial sarcoma and Ki is positive in 20 % of tumor cells nuclei, the margins were involved in the first surgery, and the patient is young. The patient has prescribed six cycles of adjuvant chemotherapy every three weeks, Doxorubicin 60 mg/m^2^, and Ifosfamide 5000 mg/m^2^. Mesna 600, 2500, and 1250 mg/m^2^ were given before, with, and after Ifosfamide, respectively. The patient was reassessed after the third cycle of chemotherapy and after three months after the second surgery. The patient ambulates freely, and the sensation is completely normal, and muscle power is 4/5.

## 3 Discussion

A sarcoma is a malignancy that arises in the tissue of the mesenchymal lineage, including muscles, bones, and adipose tissue [1]. They account for only of 1 % of human malignancies [1]. STSs are of mesenchymal origin that arise anywhere in the body, being the extremities the most common site for primary tumors, which account for 60 % of STS [7], [8]. The name synovial is a misnomer in which they don't originate from the synovium, but from the mesenchymal cells [13], [14]. Differentiating Synovial Sarcoma of the peripheral nerve (SSPN) from other malignant peripheral nerve sheath tumors (MPNST) is difficult due to many similarities in imaging findings, clinical features, and histology [15]. However, in 1987, Turc-Carel et al. [16], [17] showed the synovial sarcoma translocation gene. SS18 gene on chromosome 18 to homologous gene on the X chromosome in synovial sarcomas. This molecular pathology considers now the golden standard for differentiating and diagnosing synovial sarcoma. Treatment is individualized based on the patient's characteristics. However, the mainstay of treatment in synovial sarcoma is surgical excision with chemotherapy and/or radiotherapy based on tumor characteristics [18]. Long term follow up studies showed that pre-operative chemoradiation and postoperative chemotherapy with a Doxorubicin-based regimen improve local control and disease-free survival and overall survival [19]. As was mentioned before, intraneural ganglion cysts most commonly involve the peroneal nerve at the fibular neck as like synovial sarcoma [10]. Magnetic resonance imaging (MRI) is the gold standard diagnostic imaging for both synovial sarcoma and intraneural ganglion cysts. However, tumors smaller than 5 cm usually show homogenous enhancement and can be mistaken for benign tumors [11]. Hence, a biopsy should be done prior to surgery to avoid misdiagnosis [20]. There are some limitations to this study, which do not provide a complete picture of the patient's condition at the end of it. This study was written at the time the patient was receiving chemotherapy postoperatively.

## 4 Conclusion

Even though it is extremely rare, synovial sarcoma of the lower extremity should be considered when a painful swelling of the lower leg is associated with a long duration of symptoms. Such lesions are best managed by surgical excision and histopathology examination.

## Consent

Written informed consent was obtained from the patient for the publication of this case report and accompanying images.

## Ethical approval

Ethical approval is exempt/waived at our institution.

## Funding

There were no sponsors for this case report.

## Guarantor

Abdulaziz Saleh Almodumeegh.

## Research registration number

This case report is not a “First in Man” and therefore does not require any registration.

## CRediT authorship contribution statement

All the authors were involved in the writing of this case report.

## Declaration of competing interest

There are no conflicts of interest in the creation of this case report as declared by the authors.
